# Pilot Study of Intensive Trismus Intervention Using Restorabite™ During Unilateral Adjuvant Radiation for Head and Neck Cancer

**DOI:** 10.1007/s00455-024-10668-4

**Published:** 2024-02-16

**Authors:** Emma Charters, Jamie Loy, Raymond Wu, Kai Cheng, Masako Dunn, Sarah Davies, Jonathan Clark

**Affiliations:** 1 Department of Head and Neck Surgery, Chris O’Brien Lifehouse, 119-143 Missenden Road, Sydney, NSW 2050 Australia; 2 School of Health Sciences, Faculty of Medicine and Health, The University of Sydney, Sydney, NSW Australia; 3 Department of Radiation Oncology, Chris O’Brien Lifehouse, Sydney, NSW Australia; 4 Royal Prince Alfred Institute of Academic Surgery, Sydney Local Health District, Sydney, NSW Australia; 5 Sydney Medical School, Faculty of Medicine and Health Sciences, The University of Sydney, Sydney, NSW Australia

**Keywords:** Oral neoplasm, Trismus, Radiation oncology, Surgical oncology, Medical device

## Abstract

Trismus commonly arises after surgery for head and neck cancer (HNC) and its severity is potentiated by postoperative radiotherapy. While the benefit of trismus rehabilitation after surgery and radiotherapy is well established, the evidence during radiotherapy is less clear. This may be due to poor adherence to trismus exercises secondary to acute mucositis. This study assessed the feasibility of using a novel trismus device during adjuvant radiotherapy for HNC in patients with acute postoperative trismus. Prospective single-arm cohort feasibility study. Eligible patients had undergone surgery with curative intent for HNC, planned for adjuvant radiotherapy, and were suitable for trismus rehabilitation. Participants completed a 10-week exercise program using a novel jaw stretching device. Study outcomes were adherence, maximal incisal opening (MIO), and trismus-related function and quality of life scores, assessed at baseline, 10 weeks, and 6 months after commencing exercises. Nine patients diagnosed with trismus after primary surgery were recruited. The mean increase in MIO at 10 weeks was 7.8 mm (range −4 to 15 mm, *p* = 0.03), and at 6 months was 10.6 mm (range 1–26 mm, *p* = 0.03). Significant improvements were observed in trismus-related quality of life (Gothenburg Trismus Questionnaire; *p* = 0.04). Adherence to the exercises was 100% in week 1–2, 67% in weeks 3–6, and 100% at 10 weeks (1 month post radiation). This study demonstrates the feasibility of using a novel jaw stretching device during adjuvant radiotherapy. Further evaluation is warranted to assess the effectiveness of early intervention and prevention of trismus during HNC radiotherapy.

**Level of Evidence: IV**

## Introduction

Trismus, defined as a maximum interincisal opening (MIO) <35 mm [1], is reported to occur in 28–52% of patients treated for head and neck cancer (HNC) [2–4]. There are multiple mechanisms which contribute to the development of trismus following surgery and/or radiotherapy. Acutely, patients may develop inflammation in the temporomandibular joint, pain from mucositis, and spasm in the muscles of mastication, while chronic trismus is usually due to fibrosis [5–7]. Trismus can negatively impact on health-related quality of life (HRQOL) due to changes in facial appearance and difficulties with speech [8–10], oral hygiene, dental care [11, 12], eating, and swallowing [9, 10]. This may then lead to social anxiety, loss of dentition, and chronic malnutrition [13]. Prophylactic swallowing exercises and education have been shown to improve dysphagia-specific HRQOL scores in HNC patients [14], however, a recent systematic review demonstrated no significant difference in MIO between the intervention and control arms of any individual study, where a jaw stretching device was used to prevent trismus during HNC radiotherapy or in the first 12 months post treatment [15].

It is unclear whether this is due to poor adherence or ineffectiveness of the intervention itself. It is possible that several factors are contributing. Firstly, there is a lack of empirical evidence for the commonly used exercise regimen, which is based on hamstring exercises [8, 9, 16]. Secondly, these devices may be difficult to use in the setting of radiation mucositis due to the large size of the mouthpieces. This is supported by Melchers et al. [17], who looked at a range of factors influencing TheraBite™ exercise adherence in patients. They found that patient motivation to exercise and perceived effect had the strongest influence on adherence but ill-fitting mouthpieces were also a problem [18]. Thirdly, commonly studied, commercial devices such as the TheraBite™ and Dynasplint™ rely on the patient deciding on the appropriate level of force. While the force can be adjusted by the patient, the actual force applied to the jaw has not been quantified and it may not be consistent. In patients with underlying bone pathology due to tumour or osteoradionecrosis, there is a risk of fracture due to the force exceeding the bone’s elastic limit. Conversely, if the force applied is insufficient, therapeutic gains may be minimised.

We recently published a pilot study using a novel jaw stretching device (Restorabite™) in HNC patients with established trismus after surgery and/or radiotherapy [17]. The device allowed for a prescribed maximum force, individualised to the patient by their treating physician to minimise the risk of complications. The intervention was associated with statistically and clinically significant improvements in MIO and in trismus-related quality of life. The aim of the present study is to explore the feasibility of using Restorabite™ early in the postoperative period and during adjuvant radiotherapy, in order to minimise the synergistic effect of radiotherapy on surgery-induced trismus. We hypothesised that because the mouthpiece is smaller, and the force applied does not rely on the efforts of the user, that it may have a high level of adherence in the setting of postoperative radiotherapy.

## Materials and Methods

Ethical approval was obtained from the Sydney Local Health District Human Research and Ethics Committee (HREC Protocol X20-0404/LH20.092) and all participants provided written informed consent. Patients were recruited following surgery and prior to commencing radiation therapy for HNC between September 2021 and September 2022.

### Participants

Participants were eligible if they met the following criteria: (a) over 18 years of age, (b) planned to undergo adjuvant radiotherapy following primary surgery for HNC, (c) postoperative trismus (MIO <35 mm), (d) approved for intensive trismus rehabilitation by their treating oncologist. Potential participants were identified through referral to the Speech–Language Pathology service, contacted by the principal investigator (EC), and invited to participate in the study.

### Surgery

All patients underwent curative-intent surgery aiming for R0 resection at the primary site and nodal basin as determined by their surgical oncologist.

### Radiation Therapy

All patients were deemed suitable for adjuvant radiotherapy by a multidisciplinary team. This was delivered in conventional fractionation to a dose of 60–66 Gy using volumetric-modulated arc therapy (VMAT) with custom thermoplastic mask immobilisation and daily image guidance. Patient’s with grossly involved margins were boosted to 70 Gy and concurrent chemotherapy strongly considered for involved surgical margins or extranodal extension.

### Purpose-Built Jaw Stretching Device

**Fig. 1:**
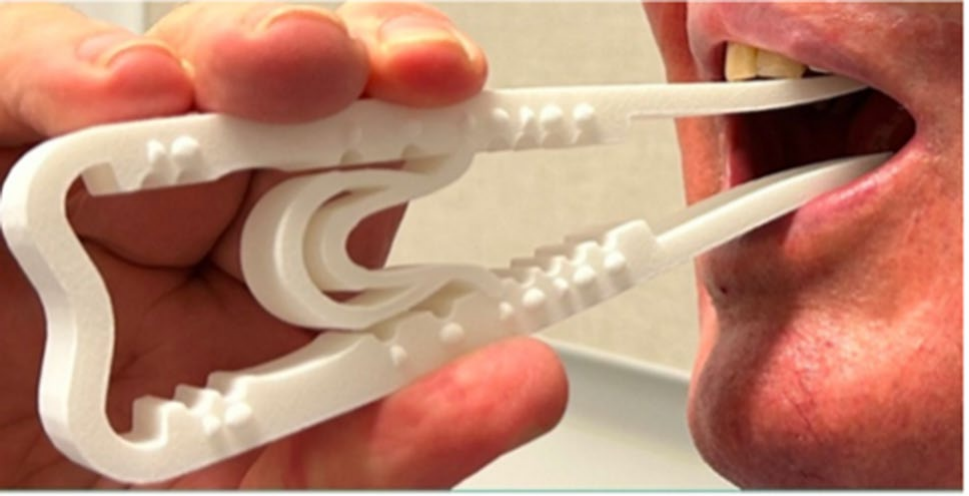
Participant using Restorabite™. Participant using Restorabite™ for a passive range of motion stretch in position 5 (maximal force in this position is 45 N)

**Fig. 2:**
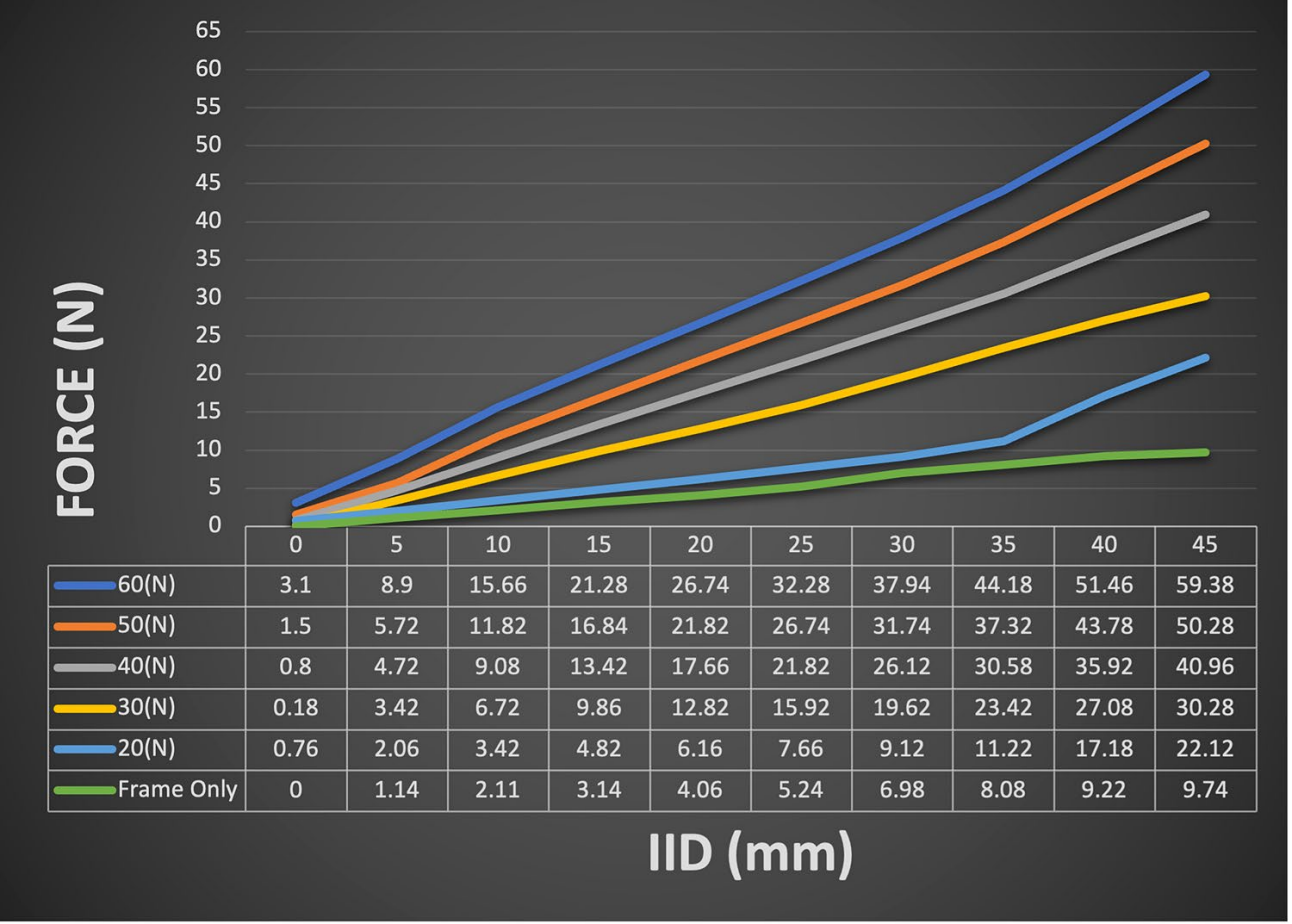
Graduated displacement forces of seven calibrated levels of resistance. N = Newton. Coloured lines represent the force achieved by the frame only (green) and 5 of the insert positions from the least resistance (light blue) to the most resistance (dark blue). This figure demonstrates the increase in force applied to the jaw as the displacement (measured in mm) increased/participant bit down. This force can be modified depending on which position the insert is in

The device piloted in this study is the same purpose-built trismus device used in a previous study (Fig. 1) [17]. Its design allows the participants to alter the level of difficulty over ten calibrated settings with maximal force constraints prescribed by their treating physician (Fig. 2). The force for the frame only, and then the insert in each position has been regulated using the ‘Universal Testing Machine’ from 0 to 45 mm displacement. The maximum force for the frame only is 10 Newtons (N), the insert can then be inserted in position 1 through 9, with a maximum force of 60 N in position 9. This allows larger forces to be used to optimise therapeutic gains, while remaining beneath a safe force limit to reduce the risk of stretching-related injuries [19]. Participants were provided with verbal and written instructions detailing how to use the device and the frequency of their treatment regime.

### Trismus Treatment

All participants underwent a 10-week individualised program using the jaw stretching device for 12 min to allow direct comparison with other literature. The initial session with the speech–language pathologist was scheduled before the commencement of radiation and included strategies to aid adherence to the intervention, individualised goal setting, and an introduction to how to progressively increase perceived effort (using a patient-rated perceived effort of 6–7/10 on the OMNI scale) [20]. The OMNI scale is a picture system used to rate perceived exertion for an activity between 0 (extremely easy) and 10 (extremely hard). Participants were encouraged to stretch proactively prior to and in the early weeks of radiation, then continue using the device at a reduced intensity once toxicities such as mucositis occurred. The study intervention was conducted by clinicians who had been trained, and then who followed the principles of exercise physiology described in the Amplified Resistance Kinetics of the Jaw (ARK J) Jaw Program [21]. The Ark-J Program is a course teaching an evidence-based approach to evaluate and treat trismus. Specifically, the participant is instructed to use the trismus device to achieve a level of difficulty that was challenging but did not elicit pain. The participants could modify the intensity either by changing the level of force or the duration of the exercise based on their individual circumstances. Participants completed daily exercises on their own and weekly consultations with the speech–language pathologist. Patients were advised not to exceed 12 min of stretching per day for the duration of the study. Twelve minutes was selected in order to compare to other exercise regimens using other trismus devices such as Therabite^®^, which typically uses a 30 s × 5 repetitions × 5 sets per day regimen (total 12.5 min). The program involved the following sequence of exercises:

- Warm up: gentle mobilisation of the jaw and massage
- Passive stretching with the jaw stretching device: The device is inserted between the participant’s teeth or gums, stretching the jaw open. The force is adjusted by the patient by increasing the level of force applied or the stretch duration and changing the position of the device in the mouth. Stretching duration was determined by the patient, however, the total stretching was 12 min per day. If the patient experienced pain, they either lowered the intensity or reduced the stretching duration.
- Active exercise with the jaw stretching device: With the device between their teeth or gums, the participant bites down to engage muscles of mastication. Use of sugar-free chewing gum was also encouraged for active engagement of the muscles of mastication.
- Participants could also elect to conduct passive-active cyclic exercises for 2–3 min. This involved starting with a passive stretch until they started to perceive discomfort, transitioning to biting down for several seconds, and then return to the passive stretch.

After the 10-week intervention period, participants transitioned to a maintenance program. For those who no longer were classified as having trismus, they were encouraged to continue to measure their mouth opening 1–2 times per week and recommence stretching if they saw a reduction in their mouth opening measure. For those who had persistent trismus, they were encouraged to continue stretches.

### Outcomes

Outcomes of interest were adherence, adverse events, MIO, and patient-reported function and HRQOL. All outcomes were assessed by speech–language pathologist at baseline and repeated at 10 and 26 weeks post completion of the intervention.

There are no validated adherence measures for this population. Adherence to trismus exercises was rated as (a) did not do any trismus exercises, (b) exercised less than recommended, (c) exercised as recommended, or (d) exercised more than recommended.

MIO was assessed using the TheraBite^®^ Jaw Range of Motion Scale placed between the upper and lower central incisors. If the patient was missing dentition, the measure was taken from the maxillary to mandibular gingiva, subtracting 10 mm for each row of missing dentition [22].

Trismus-related QOL was assessed using the Gothenburg Trismus Questionnaire (GTQ), a validated patient-reported questionnaire that measures the impact of trismus on the patient’s daily life [23]. Swallowing and speech was assessed using the MD Anderson Dysphagia Inventory (MDADI) [24], Eating Assessment Tool—10 (EAT-10) [25], and the Speech Handicap Index (SHI) [26]. The MDADI, composed of 20 questions, investigates how swallowing affects an individual’s global, physical, emotional, and functional aspects. The composite score ranges from 20 to 100, where 20 indicates low and 100 indicates high swallowing function. The EAT-10 calculates a score from 0 to 40, where a higher score denotes worse swallow function. Finally, the McGill Questionnaire (short form) (MGQSF) was completed by the patient to describe the type and severity of sensation associated with their pain including an overall pain intensity measure [27]. These validated patient-reported outcome measures were used to examine the impact that intervention for trismus might have on quality of life and physical function.

### Statistical Analysis

Statistical analysis was carried out with system software R i386 3.2.2 (The R Foundation for Statistical Computing). Changes in pre- to post-trismus intervention scores for continuous variables (composite scores for the Gothenburg, EAT-10, MDADI, SHI, and MGQSF) were calculated using student *t*-tests for univariate analyses. Generalised estimating equations (GEEs) were used to model MIO adjusting for baseline effect. Descriptive statistics were used to describe the demographics and clinical details of the participants.

## Results

### Participants

Nine patients (seven male and two female), ranging in age from 44 to 75 years (mean 56.1 years), were recruited. Complete pre- and post-intervention speech pathologist assessments were available for all nine patients. Their demographics, treatment, and clinical details are outlined in Table 1. All participants had trismus prior to commencing postoperative radiotherapy as a result of primary surgical tumour management (transoral robotic surgery = 3, parotidectomy = 3, mandibulectomy = 2, and partial glossectomy = 1) and commenced trismus therapy between 3 and 6 weeks post surgery (mean 4.8 weeks).

**Table 1:** Patient’s age, gender, tumour site, TNM classification, and treatment details

Participant number	Age	Gender	Tumour and site	Tumour classification AJCC 8th edition	Time post treatment (weeks)	Radiotherapy dose (Gy/#)	Laterality
1	59	M	Parotid SCC	pT1N0M0	6	60 Gy/30#	Ipsilateral parotid
2	44	M	Tongue SCC	pT2N1M0	4	70 Gy/35#	Ipsilateral oral cavity and bilateral neck
3	59	M	Left cheek cutaneous SCC	pT4bN0M0	4	60 Gy/30#	Ipsilateral parotid, upper neck and base of skull
4	53	M	Tonsil SCC	pT1N1M0	6	60 Gy/30#	Ipsilateral tonsil and neck
5	43	M	Parotid SCC	pT3N0M0	3	60 Gy/30#	Unilateral parotid
6	52	M	Mandible SCC	pT4N1M0	6	60 Gy/30#	Unilateral oral cavity and neck
7	53	F	BOT SCC	pT2N1M0	3	60 Gy/30#	Ipsilateral neck
8	63	F	Mandible SCC	pT4N0M0	5	66 Gy/33#	Ipsilateral oral cavity and neck
9	69	F	Tonsil SCC	pT1N1M0	6	60 Gy/30#	Ipsilateral neck

*SCC* squamous cell carcinoma, *Gy* Gray (radiation), # fractions, *BOT* base of tongue

Time post treatment: time in weeks after surgical date participant commenced trismus therapy

### Adverse Events

No participants experienced any adverse events as a direct result of the intervention.

### Adherence

All nine participants completed the intervention period of 10 weeks and were retained for the duration of the trial. A range of techniques were used to increase adherence including scheduling trismus therapy after administering analgesics, using saliva substitutes during stretching, and carefully positioning the trismus device to avoid areas affected by mucositis.

**Fig. 3:**
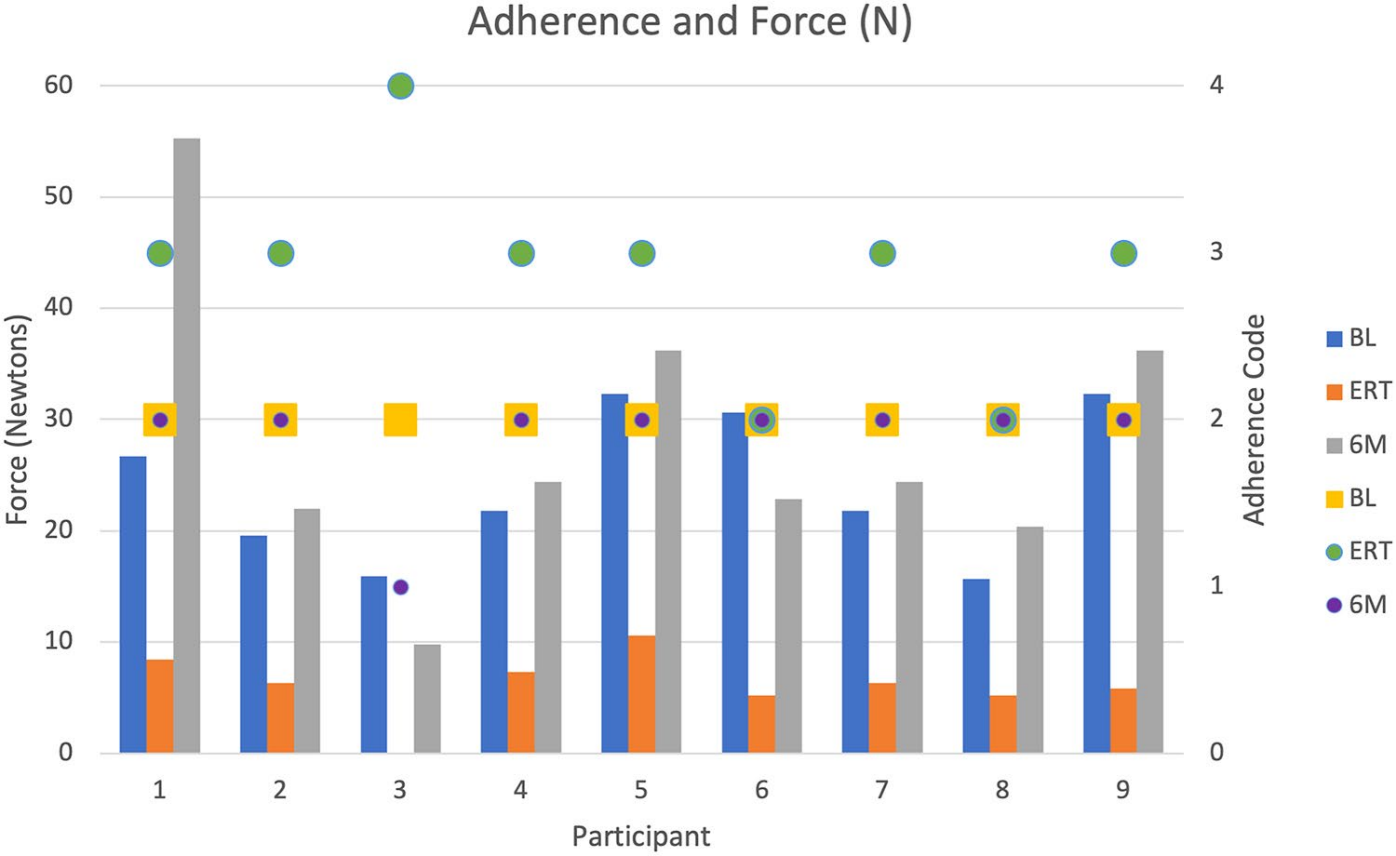
Adherence and intensity for each participant. Adherence to exercise and force applied in passive range of motion stretching. Adherence (A) A1: more than recommended, A2 = as recommended, A3 = less than recommended, A4 = no exercise. *BL* baseline, *ERT* end of radiation therapy, *6M* 6 months after starting trismus therapy. Columns indicate force (measured in Newtons N), markers indicate adherence

In the first 3 weeks of radiation therapy, all nine participants exercised as recommended. In weeks 4–6, six participants (67%) exercised less than recommended, two (22%) exercised as recommended, and one (11%) did not do any trismus exercises. During weeks 7–10, eight participants (89%) exercised as recommended and one participant (11%) exercised less than recommended (Fig. 3). No participants exercised more than recommended during the intervention period.

### Force

Force for passive range of motion exercises was highest at the start of the intervention with a mean of 24.1 N (range 15.7–32.3 N) and dropped to a mean of 7.2 N (range 5.2–10.6 N) by week 6. By the conclusion of the study, the mean intensity was 29.3 N (range 9.8–55.3 N).

### Maximal Incisor Opening (MIO)

The mean MIO prior to intervention was 23.0 mm (range 14–34 mm). According to the criteria described in Dijkstra et al. [1], two participants were classified as having mild trismus, six had moderate trismus, and one severe trismus. At the completion of the 10-week intervention program, the mean MIO was 30.8 mm (range 23–43 mm). The mean change in MIO after 10 weeks of therapy was 7.8 mm (*p* = 0.03, Table 2) and at 6 months was 10.6 mm (*p* = 0.03). At 6 months, five participants (59%) no longer met the diagnostic criteria for trismus. Adjusting for the effect of age, T category, sex and baseline MIO, the estimated increase in MIO was 0.4 mm per week over 6 months (*p* = 0.003) (Table 2).

**Table 2:** Composite GTQ, MDADI, MGPSF, EAT-10, SHI score, mean value, standard error (SE) for participants at baseline (prior to commencing trismus therapy), after completing 10 weeks of trismus therapy using Restorabite™, and at 6 months of follow-up

	Prior to starting trismus therapy	10 weeks post trismus therapy	6 months post trismus therapy	Difference (baseline to 6 months)	*p*	*Cohen’s d*	95% CI
	Mean (SE)	Mean (SE)	Mean (SE)				
MIO	23.0 ± 9.4	30.8 ± 7.1	33.6 ± 7.7	10.6	0.03	1.3	[−3.3 to 5.8]
GTQ	40.5 ± 20.6	24.1 ± 14.6	19.3 ± 11.9	−21.2	0.04	1.0	[−9.2 to 11.2]
MDADI	63.3 ± 18.3	73.1 ± 4.0	72.2 ± 2.9	8.9	0.5	0.5	[−8.6 to 9.6]
McGill	7.2 ± 6.5	12.5 ± 1.5	2.7 ± 2.1	−4.5	0.1	0.6	[−2.6 to 3.9]
EAT-10	17.3 ± 12.2	28.4 ± 4.5	14.0 ± 3.5	−3.3	0.4	0.3	[−5.8 to 6.4]
SHI	29.0 ± 28.0	21.5 ± 12.4	32.4 ± 6.7	−3.4	0.8	0.1	[−13.8 to 14.0]

*Cohen’s d* = effect size

### Patient-Reported Outcomes

Patient-reported outcomes at 10 weeks and 6 months are shown in Table 2. Statistically significant improvements were observed for the composite scores of the GTQ (*p* = 0.04). All other outcomes (MDADI; *p* = 0.5, McGill pain; *p* = 0.1, EAT-10; *p* = 0.4, SHI; *p* = 0.8) were not significant at 6 months post trismus intervention.

## Discussion

This study demonstrates the feasibility of the use of a novel trismus device starting between 3 and 6 weeks post surgery and before commencement of adjuvant radiotherapy. We observed satisfactory levels of adherence to this early and intensive intervention, and improvements in MIO and trismus-related quality of life in patients despite the effects of radiotherapy (Table 3).

**Table 3:** GTQ, MDADI, MGPSF, EAT-10, SHI GEE analysis for participants prior to, and 6 months trismus therapy with the hospital-manufactured trismus device

	Estimate	SE	*p* value
MIO (low score indicates greater dysfunction)			
Time point (baseline vs. **6 months**)	9.3	1.8	**0.003**
Age (per year)	0.4	0.1	**0.003**
T stage (early vs. **late**)	0.3	0.1	0.1
Gender (**M** vs. F)	0.00	1.6	1.0

Bold values indicate the significant categorical variable

Estimates: Time: change in score between baseline at 6 months, Age: change in score per of age, T stage: scores of late stage (T3 or T4) tumours compared to early stage (T1 or T2) tumours, gender: scores of males compared to females

It is the first study to our knowledge that explores the use of a trismus device within 6 weeks of surgery for patients, intervening prior to the onset of radiation fibrosis. MIO improved over the 10 weeks intervention on average by 7.8 mm and no patients experienced worsening trismus from baseline prior to radiotherapy to 6 months post treatment. Additionally, the study demonstrated that patients could maintain the gains achieved up to 6-months, which is a critical time for trismus to develop [28].

Adherence to exercises is a critical component of any rehabilitation program. Exercise adherence holds additional complexity during radiation given the toxicities experienced by patients which may act as a deterrent [17]. Prior studies have found that suboptimal adherence to exercise regimens prevents therapeutic gains [17, 29]. Studies which explore facilitators and barriers to exercise regimens are needed to understand how best to support patients adhere to the exercises recommended. The nine participants demonstrated variable adherence during radiation. While complete adherence was seen at baseline and 10 weeks, most participants (7/9, 78%) exercised less than recommended in weeks 3–6 of radiation. This is not unexpected given that the toxicities associated with radiation therapy peak at this point making any oral exercise challenging. Future studies could explore whether reducing the stretching force or duration during the latter half of radiotherapy may help to maintain exercise adherence while still achieving some positive impact on trismus.

Principles for evaluating adherence are explored in Hawley-Hague et al. [30]. They describe four criteria for measuring adherence; (a) completion/retention, (b) attendance, (c) duration adherence, and (d) intensity adherence. For the purposes of this study, adherence was recorded weekly by the patient based on the question ‘did you do your jaw exercises:’, (a) not at all, (b) less than recommended, (c) as recommended, or (d) more than recommended. The force (calculated from the MIO) and exercise duration were also recorded by the patient.

Previous research suggests that stretching devices that allow both passive and active stretching (such as Therabite^®^) are superior to static tools, such as stacked tongue depressors which provide passive range of motion exercises only [18]. However, the cost of commercially available dynamic trismus devices can be prohibitive, and there are risks of fractures to the jaw and dentition [9, 17, 31]. This may lead to reluctance to use trismus devices, particularly early post-surgery due to concerns about bone non-union in the reconstructed jaw and the risk of dental or mandibular fracture post radiotherapy. One of the key findings of this study is that beginning trismus intervention between 3 and 6 weeks post surgery was found to be safe with no adverse events reported. This is particularly important for participants whose surgical intervention involved a mandibulectomy where conventional wisdom has recommended avoiding applying pressure until sufficient healing occurs. This suggests that starting trismus treatment early may be feasible when the force can be regulated reliably.

Understanding the optimal force for safe trismus rehabilitation may lead to better functional and quality of life outcomes [16, 29, 32]. This is likely to be patient specific and computational methods could be used to model the jaw biomechanics incorporating the individual’s unique factors such as bone density to estimate of how much force the jaw can safely withstand during the trismus exercises, thus maximising their rehabilitation capacity prior to beginning radiation and the onset of mucositis and fibrosis.

As a feasibility study, this paper has several limitations. The low participant number compromises the reliability of the findings. Additionally, the lack of a control group limits the internal validity of changes in MIO and patient-reported outcomes, which may have improved without intervention. The study population was relatively heterogeneous based on primary tumour site and treatment administered. This introduces a variety of complex causal factors for the development and severity of trismus, including surgical scarring, postoperative oedema, muscle denervation, and radiation-induced fibrosis. Although participants were given a maintenance program to continue, there is substantial risk that the improvements obtained by intensive intervention may diminish over time, and therefore a longitudinal analysis is planned in a larger patient group to enhance the generalisability of the findings. A final note is the lack of assessor or participant blinding, particularly given the author’s role in the design and patent of this device.

## Conclusion

This study demonstrates the feasibility of applying a novel trismus device and exercise regimen in patients with acute trismus during postoperative radiotherapy for HNC. Further research is necessary to investigate its efficacy in larger cohorts with suitable controls. The use of an affordable device that allows for the precise calibration of the applied force could lead to safer, more widely accepted, and cost-effective treatments for trismus.

## Data Availability

The datasets generated during and/or analysed during the current study are available from the corresponding author on reasonable request.
